# A Non-Destructive Culturing and Cell Sorting Method for Cardiomyocytes and Neurons Using a Double Alginate Layer

**DOI:** 10.1371/journal.pone.0042485

**Published:** 2012-08-03

**Authors:** Hideyuki Terazono, Hyonchol Kim, Masahito Hayashi, Akihiro Hattori, Fumimasa Nomura, Tomoyuki Kaneko, Kenji Yasuda

**Affiliations:** 1 On-chip Cellomics Project, Kanagawa Academy of Science and Technology, Kawasaki, Kanagawa, Japan; 2 Division of Biosystems, Department of Biomedical Information, Institute of Biomaterials and Bioengineering, Tokyo Medical and Dental University, Chiyoda-ku, Tokyo, Japan; University of Cincinnati, United States of America

## Abstract

A non-destructive method of collecting cultured cells after identifying their *in situ* functional characteristics is proposed. In this method, cells are cultivated on an alginate layer in a culture dish and released by spot application of a calcium chelate buffer that locally melts the alginate layer and enables the collection of cultured cells at the single-cell level. Primary hippocampal neurons, beating human embryonic stem (hES) cell-derived cardiomyocytes, and beating hES cell-derived cardiomyocyte clusters cultivated on an alginate layer were successfully released and collected with a micropipette. The collected cells were recultured while maintaining their physiological function, including beating, and elongated neurites. These results suggest that the proposed method may eventually facilitate the transplantation of ES- or iPS-derived cardiomyocytes and neurons differentiated in culture.

## Introduction

Embryonic stem (ES) or induced Pluripotent stem (iPS) cells are widely expected to be used in clinical therapeutics for transplantation [1], [2], [3], [4] or as drug screening tools [5], [6], [7], [8]. For each passaging of cells, cell lines are usually detached from the culture dish using collagenase or trypsin, which degrades the extracellular matrix or proteins. This is harmful to cells, so fragile cells, such as cultured primary neurons, cannot be recultured.

Recently, Okano et al. have developed techniques that enable the detachment of cells from culture dishes without using digestive reagents [9]. A temperature-dependent polymer, poly (N-isopropylacrylamide [PIPAAm]), changes its hydrophilic/hydrophobic properties as the temperature changes. PIPAAm is hydrophobic at 37°C and hydrophilic at 20°C, so cells on a PIPAAm-coated culture dish can be detached without destroying the extracellular matrix and intercellular connections, such as tight junctions. These methods therefore yield cell sheets that maintain their intercellular connections. Using this technology, cardiac tissue can be grown by stacking mono-layered cardiac cell sheets [10]. In another study, cell sheets made from corneal epithelial stem cells have been investigated for cornea therapeutics [11].

However, single cells with specific properties cannot be collected by this method because temperature cannot be spatially controlled with micrometer resolution. Moreover, dispersed cultured cells may have variable physiological properties and may not be homogeneous. To ensure that the physiological properties of cells are truly homogeneous, it is necessary to develop a method to measure the phenotypes of single cells in culture dishes and then collect them individually without perturbation of the cells.

Alginate is a useful polymer for use as a culturing scaffold because it is non-perturbing to cells and has been used for 3-D cultivation [12], [13], [14], [15], 3-D printing [16], and nanosheets [17]. It has another interesting property: it can be gelled by replacing sodium ions with calcium ions and restored to a sol state by removing the calcium ions from the gel by chelation. Furthermore, solation can be regulated by spot application of chelate solution using a micropipette and can be controlled with a spatial resolution on the order of forty microns.

In this paper, we describe a technique that enables single cultured cells with particular phenotypic characteristics to be selected from a culture dish using spot melting of calcium alginate. Primary hippocampal neurons, cardiomyocytes derived from human ES cells, and cardiomyocyte clusters derived from human ES cells were collected nondestructively from a culture dish after identifying their phenotypes *in situ*.

## Methods

### Neuron preparation and cultivation

Dispersed cultures of hippocampal cells were prepared from 18-day-old embryos (E18) of Wistar/ST rats (Saitama Experimental Animals Supply) in accordance with the National Institute of Health guidelines for laboratory animal care and safety. The hippocampal formation was dissected from anesthetized animals in ice-cold Hanks balanced salt solution (HBSS) and then treated with 0.25% trypsin (Wako) and 0.01% DNase I (Sigma) at 37°C for 30 min. After inhibiting trypsinization by adding horse serum, cells were centrifuged at 150× g for 5 min. The pelleted cells were dispersed in 2 mL Neurobasal (Invitrogen Neurobasal medium) supplemented with 2% B-27 (Invitrogen) and 1% penicillin-streptomycin at 37°C. For primary cultures, neurons and glial cells were plated onto a 35-mm culture dish coated with poly-L-lysine (Iwaki) at a cell density of 1.0×10^5^ cells/cm^2^ at 37°C in a humidified 5% CO_2_ and 95% air atmosphere.

### Cardiomyocytes derived from human ES cells: preparation and culturing

Cardiomyocyte clusters derived from human ES cells were purchased from Cellartis AB (Sweden), and cell suspensions of cardiomyocytes were prepared from the cell clusters. The cell suspensions were trypsinized for 5 min at 37°C. Trypsinization was stopped by adding ten volumes of a culture medium (DMEM low glucose with 10% fetal bovine serum and 1% penicillin-streptomycin) and centrifuging at 150× g for 5 min.

### Fabrication of a non-destructive cell collection dish for cardiomyocyte clusters and single cells

First, 80 µL of 1.5% sodium alginate was transferred to a 35-mm culture dish or multi-electrode array dish. The sodium alginate (300–400 cps, Funakoshi Co., Ltd., Tokyo, Japan) was spin-coated onto the culture dish with a spin-coater (model 1H-DX2, Mikasa Co., Ltd, Tokyo, Japan) at 2,000 rpm for 5 sec, then at 3,000 rpm for 10 sec, and then dried. After that, the sodium alginate was gelled by applying 1.5% CaCl_2_ (Wako Pure Chemical Industries, Ltd., Osaka, Japan), and a calcium alginate thin layer was formed in the culture dish. Next, after washing the alginate layer three times with double-distilled water, a mixture of 3.0 mg/ml type IV collagen and 1.5% sodium alginate in the proportion of one part to two parts was made, and the mixed solution was transferred to the calcium alginate layer in the culture dish and layered by a spin-coater as above. In the case of a single layer made with a mixed solution, the extracellular matrix in the mixed solution guided pseudopods to the surface of the culture dish, preventing detachment.

### Fabrication of a non-destructive cell collection dish for primary neurons and neuronal networks

First, 80 µL of 1.5% sodium alginate was transferred to a 35-mm culture dish, which was spin-coated at 2,000 rpm for 5 sec, then at 3,000 rpm for 10 sec, and then dried. Then, the sodium alginate was gelled by applying 1.5% CaCl_2_, and a thin layer of calcium alginate was formed on the culture dish. Next, the dish was coated with poly-L-lysine hydrobolate (PLL) by the micro-contact printing (µCP) method. The mold was designed with 10×10 µm^2^ pillar arrays, and the spacing between the pillars was 2 µm with a depth of 10 µm. The mold was formed from poly-dimethylsiloxane (PDMS). PLL was dissolved in 1-mM carbonate buffer, and the mold was incubated in 200 mM of this PLL solution for 15 min and then dried. The PLL-coated mold was placed in contact with the alginate layer for 5 min and detached slowly to prevent peeling of the alginate layer.

### Detaching and transferring the cells

For detaching and transferring the cells, a glass capillary with an 0.6-mm internal diameter was heated, pulled, and fire-polished to make the final internal diameter of ∼40 µm using a puller (model: PC-10, Narishige, Tokyo, Japan) and a micro forge (model: MF-830, Narishige, Tokyo, Japan). The microcapillary was then siliconized with Sigmacote (Sigma, St. Louis, USA). To detach the cells, the capillary was filled with a culture medium containing 5 mM EDTA⋅2Na (Dojindo Laboratories, Kumamoto, Japan).

### Reculture of collected cells

The release of the medium containing EDTA from the microcapillary and the retrieval of cells was controlled by adjusting the air pressure in the microcapillary with a pneumatic manual microinjector (model: CellTram air, Eppendorf, Tokyo, Japan). The retrieved cells were then transferred to another culture dish and cultivated. The second cultivation dish was coated with PLL for the neurons and with collagen type I-C (Nitta gelatin, Japan) for the cardiomyocytes.

### Multi-channel electrode arrays (MEAs) recordings

The extracellular action potentials of cardiomyocyte clusters were measured using a 64-channel multi-channel electrode arrays (MEAs) system. MEAs were formed on a glass slide comprised of 64 ch of 50×50 µm electrodes 300 µm apart from each other and arranged in an 8×8 grid. The surface of each of the recording terminals was coated with Pt/Pt-black to reduce the impedance. The extracellular signals detected by the multiple electrodes were amplified by a 64-ch amplifier (NF corporation, Tokyo) and stored on a personal computer. A sampling rate of 10 kHz per channel was used. Signals were filtered with 2 kHz of low path filter and 1 Hz of high path filter. Data was first processed with CellAD (DITECT, Tokyo) data acquisition and conversion software and then analyzed with FlexPro.7.0 (Weisang, Germany). Extracellular signals that exceeded a threshold level set to ±5 σ were detected, where σ is the standard deviation of the baseline noise during quiescent periods.

## Results

### Fabrication of a collection dish for cardiomyocytes

**Figure 1:**
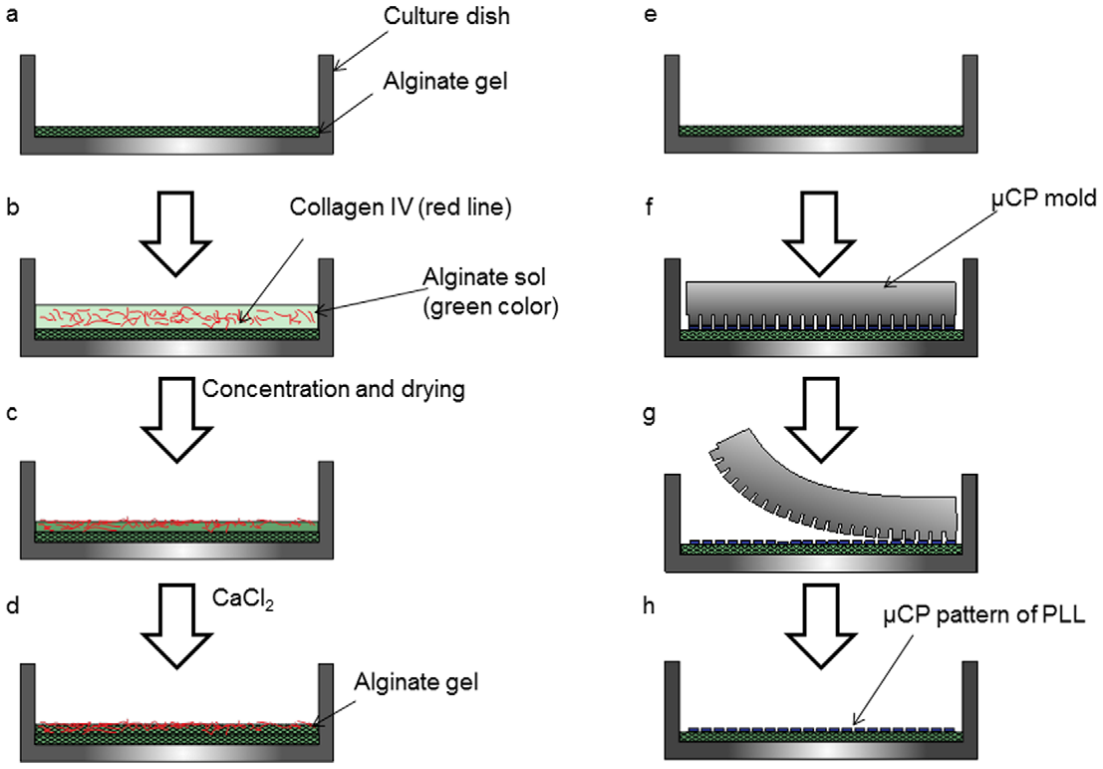
Construction of the collection dish for cardiomyocytes and neurons. First, a calcium alginate layer was formed in both culture dishes (a), (e). For the cardiomyocyte culture dish, a mixture containing a low concentration of alginate and collagen type IV extracellular matrix was layered onto the alginate sheet (b). The solution was then concentrated and dried (c), gelled with CaCl_2_, and washed three times (d). For the neuron culture dish, PLL was micro-contact printed using a PDMS stamp (f). Following incubation for 5 min under high humidity conditions (similar to those in a cell incubator), the stamp was removed from the culture dish.

First, sodium alginate was transferred to a culture dish that was coated with a spin-coater (model 1H-DX2, Mikasa Co., Ltd, Tokyo, Japan) and then dried in air. Then, the sodium alginate was gelled by ion exchange to calcium alginate following the addition of a CaCl_2_ solution (Fig. 1a). The resulting thin calcium alginate sheet was dried and washed. Next, solutions of sodium alginate and collagen type IV were mixed in the proportion of one part to two, transferred to the alginate-coated culture dish (Fig. 1b), concentrated, and dried at room temperature (Fig. 1c). The mixed solution was then gelled by again applying a CaCl_2_ solution (Fig. 1d). The resulting thin calcium alginate sheet mixed with collagen was then dried and washed.

Normally, most cells will not adhere to the calcium alginate gel layer because of its anionic composition. To obtain adhesiveness to cells, calcium alginate must be further processed by one of several methods. One method is to mix sodium alginate with an extra-cellular matrix, such as collagen [18], fibronectin, vitronectin, or laminin prior to gel formation. The other methods involve coating with cationic reagents such as polylysine (poly-L-lysine or poly-D-lysine) or bridging the arginine-glycine-aspartic acid (RGD) tri-peptide to alginate using 1-Ethyl-3-[3-dimethylaminopropyl]carbodiimide hydrochloride (EDC) [19], [20]. The alginate treatment needed to create a surface on which cells can adhere depends on the cell type. For example, cardiomyocytes can adhere to a collagen-coated culture dish. There are several types of collagen, such as I-A, I-C, I-P, III, or IV, and almost all of these collagens are kept in solution under acidic conditions and gel under neutral conditions. On the other hand, sodium alginate can be irreversibly gelled without calcium ions under acidic conditions and only collagen type IV is difficult to gel in neutral conditions. Collagen type IV is therefore the most appropriate for retaining both a reversible sol-gel transformation by chelation and adherence of cardiomyocytes to the alginate sheet. The proportion of extracellular matrix and sodium alginate is key, and studies have shown that mixing collagen type IV and sodium alginate in the proportion of one part to two parts is the optimal ratio. If the proportion of sodium alginate is too high, cells do not adhere to the sheet, and if it is too low, the alginate does not melt.

For the cardiomyocyte dish, a calcium alginate layer was fabricated under an alginate/collagen IV layer. If only an alginate/collagen IV layer is fabricated on the culture dish, cardiomyocytes adhere to the bottom surface of the culture dish through the alginate/collagen layer. Therefore, even if the alginate/collagen layer is solated by EDTA, the cardiomyocytes remain adhered to the culture dish. To solve this problem, we fabricated a layer of simple alginate, which has an anti-adhesion property, under the alginate/collagen IV layer.

### Fabrication of a collection dish for primary neurons

First, sodium alginate was transferred to a culture dish that was coated by a spin-coater and then dried in air. Next, sodium alginate was gelled by adding a CaCl_2_ solution. The resulting thin calcium alginate sheet was dried and washed (Fig. 1e). The dish was then coated with poly-L-lysine hydrobolate (PLL) using the micro-contact printing (µCP) method (Fig. 1f–g). The printing design consists of an array of 10×10 µm^2^ squares, with 2 µm between each square and a total printing area of 1 mm^2^.

Primary neurons could adhere to the alginate/collagen IV layer, but the adhesion ratio was low and did not allow the neurites to extend. We know from previous studies that primary neurons can adhere to the PLL-coated alginate dish [21] and easily enable the extension of neurites and axons. However, PLL causes insoluble alginate material by anion/cation interactions. When we applied PLL using µCP, both adhesion and neurite extension properties for neurons and the detachable property of the culture dish could be obtained. Moreover, the design of the µCP was important. If the spaces between squares were wider than 3 µm, it was difficult for neurons to extend their neurites. By the same token, if the squares were too wide, insoluble alginate covered all of the neurons and neurites and inhibited reculturing in another culture dish (data not shown).

### Collection of cultured human ES cell-derived cardiomyocyte clusters and single cells and re-culturing

**Figure 2:**
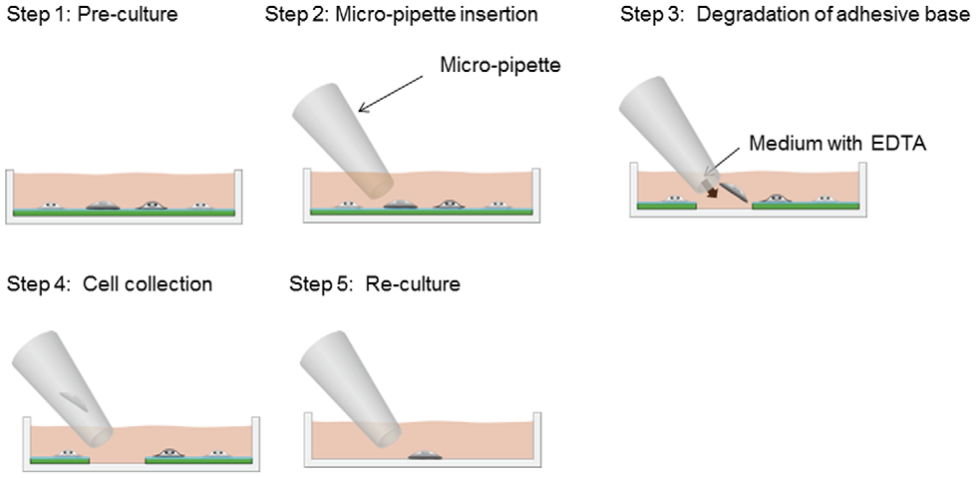
Schematic of cultured cell detachment and reculturing. In step 1, cells are pre-cultured in an alginate-coated culture dish. In steps 2–4, the alginate layer is locally isolated by applying an EDTA-containing culture medium with a micropipette, and cells cultured on the alginate are collected. In step 5, cells collected with the micropipette are recultured in another culture dish.

The steps for collecting the cultured cells are shown in Fig. 2. In step 1, cells are pre-cultured on an alginate-layered culture dish. In steps 2 to 4, the alginate layer is locally isolated by applying an EDTA-containing culture medium using a micropipette and cells cultured on the alginate are collected. In step 5, cells collected with a micropipette are recultured in another culture dish. This method enabled us to initially culture human ES cell-derived cardiomyocyte clusters and single cells in the detaching culture dish for cardiomyocytes. After a few minutes, both clusters and single cells started to adhere to the detaching culture dish. After one day, both cardiomyocyte clusters and single cells were beating regularly. After confirming that cells were beating, the medium containing EDTA was loaded using a microcapillary. Then, calcium alginate around the target cluster and single cell was immediately isolated and the target cells released from the layer. The released clusters and cells were easily collected by the pipette and cultured in another dish coated with collagen.

**Figure 3:**
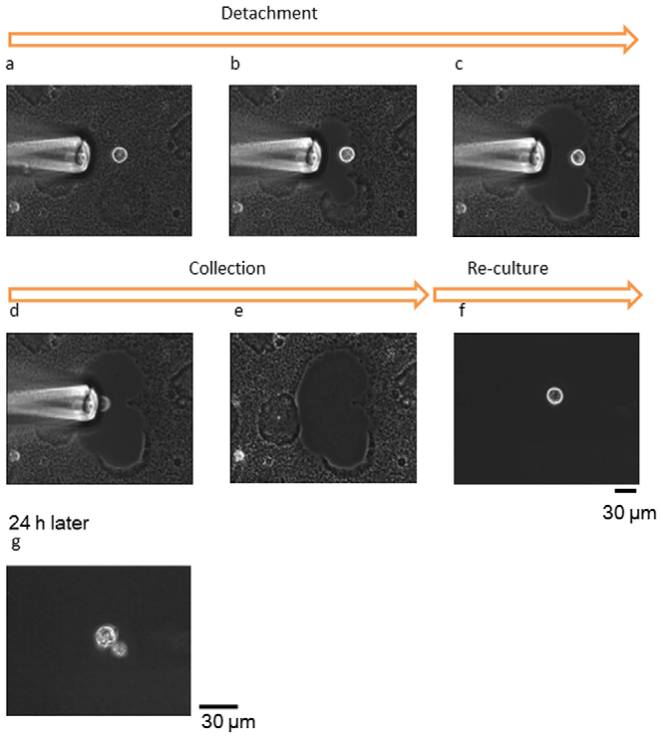
Micrographs of steps from pre-culture, detachment, and collection to reculturing of single cardiomyocytes derived from human ES cells. The time from detachment to reculturing was <2 min. During isolation, the cardiomyocytes stopped beating because the EDTA-chelated calcium ions required action potentials. After reculturing, cardiomyocytes began to beat again within a few minutes.

Figure 3 shows time course images captured from videos of pre-treatment and post-treatment. All processes, from the release of cultured cells to collection, were performed quickly (<2 min). The collected cells were re-cultured on other dishes coated with type IV collagen gel. Within a few minutes, the re-cultured cells started to beat and again adhered to the collagen-coated dish. By contrast, the cells damaged by release from the culture dish with trypsin or collagenase usually did not beat or adhere again for hours. Our method, in contrast to the conventional method, does not damage the membrane proteins of released target cells, so the cells were able to begin beating and adhering again immediately. After one day, recultured cells continued to beat in the culture dish.

**Figure 4:**
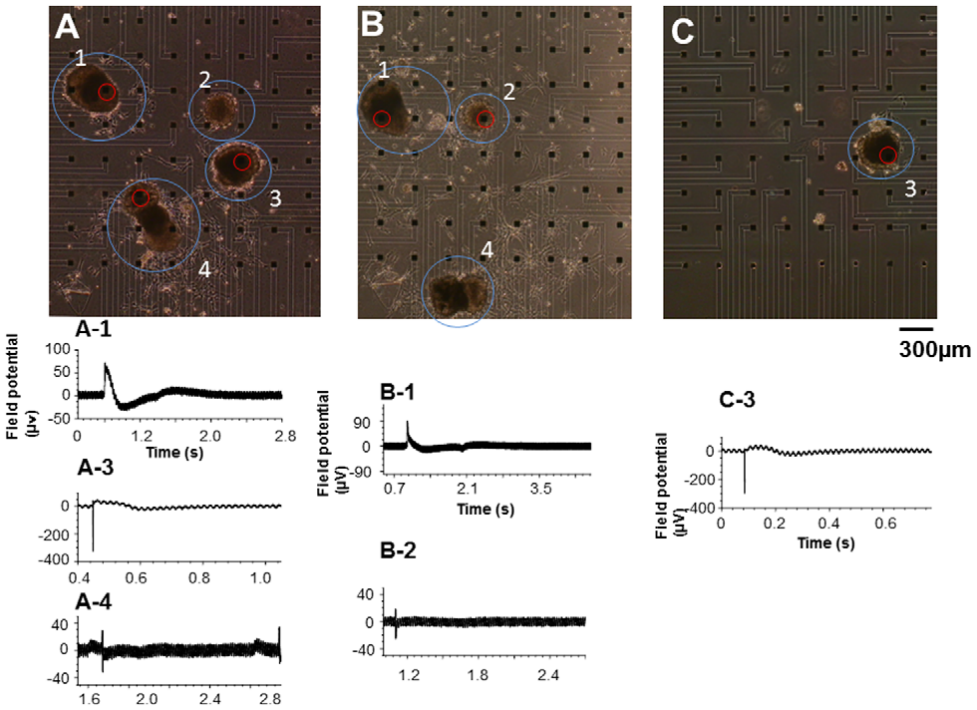
Micrographs and extracellular action potentials of cardiomyocyte clusters derived from human ES cells. Fig. A shows a micrograph of four cultured cardiomyocyte clusters derived from hES cells on the alginate thin sheet above the multielectrode array culture dish (1 day in vitro (DIV)). (A)-1 to (A)-4 are the extracellular field potentials of the cardiomyocyte clusters in (A). After the extracellular field potential measurement, the target cluster that had ventricular-type signals (cluster no. 3) was detached from the dish and recultivated on the other dish. (B) shows the remaining three clusters after the detachment of the target cluster (4 DIV). (B)-1 and (B)-2 are the extracellular field potentials. (C) shows the selected target cardiomyocyte cluster recultivated on the other multielectrode cultivation dish (4 DIV). (C)-3 is the extracellular field potential of cluster no 3. The target cluster (no. 3) was successfully picked up from the dish on which a plurality of clusters were cultivated. It retained similar characteristics of external field potential waveforms before and after the sorting procedure.

In order to demonstrate the physiological effect of treatment-induced detachment, the extracellular field potentials of cardiomyocyte clusters were measured by multi-electrode arrays and compared before and after detachment (Fig. 4). Four cardiomyocyte clusters were derived from hES cells on the alginate thin sheet above the multielectrode array culture dish. The extracellular field potentials were detected from three clusters (A1, A3, and A4). Results of waveform analysis showed ventricular-type signals emanating from A3. A3 was then detached from the dish using an EDTA-containing culture medium and recultivated on the other dish. The field potential of the recultivated A3 cluster was successfully detected on the other multielectrode cultivation dish, and the waveform of the external field potential retained similar characteristics before and after sorting (4 days in vitro: 4DIV) (Fig. 4 (C)). Moreover, the remaining three clusters (B1, B2, and B4) continued beating. The field potentials from B1 and B2 were detected and the waveforms had similar characteristics before and after sorting (4DIV). These results indicate that the detaching treatment was non-destructive and did not perturb the physiological function of the cardiomyocytes.

### Collection of cultured primary hippocampal neurons and reculturing

**Figure 5:**
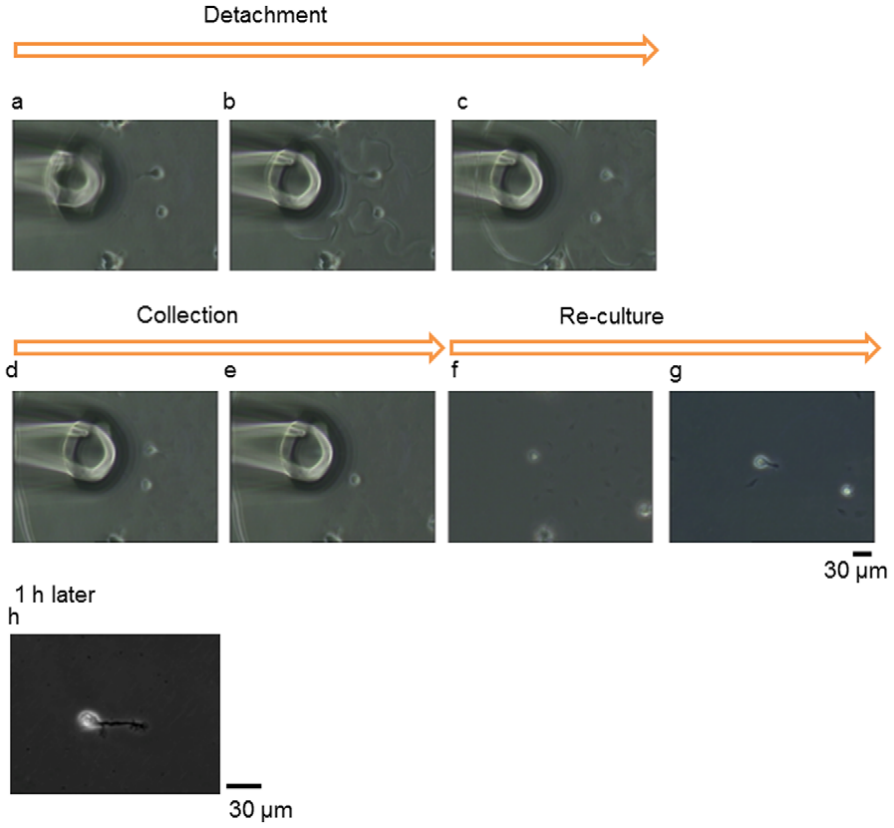
Micrographs of the steps from preculture, detachment, and collection to reculture of a single hippocampal neuron derived from rat E18. The time from detachment to reculture took <2 min, as with the cardiomyocytes. During and after isolation, the neuron did not shrink and the neurite retained its shape. After reculturing, the neuron immediately adhered to the culture dish and extended its neurite within a few minutes.

Primary hippocampal neurons were cultured in the detaching culture dish for neurons. After a few minutes, these neurons started to adhere to the detaching culture dish, and after one day, extended their neurites. After confirming that the cell neurites were extended, a medium containing EDTA was loaded using a microcapillary. The calcium alginate around the target cluster or single cell was immediately isolated, as with the cardiomyocytes (Fig. 5), and the target cells were released from the alginate layer. The released neurons were easily collected with the micropipette and cultured in another dish coated with PLL. All steps, from collection to reculturing, took <2 min. The collected neurons maintained their shapes without shrinking, and the recultivated neurons extended their neurites immediately.

## Discussion

Alginic acid is a viscous gum derived from algae and composed of β-D-mannuronate and α-L-guluronate [22]. Calcium alginate, which is a salt of alginic acid, is harmless to cells and is used as a scaffold in tissue transplantation [23]. However, cells cannot adhere to intact calcium alginate. In this study, we fabricated alginate sheets that undergo the sol/gel state transformation and are adhesive to cells. Using such a sheet, a specific cell can be collected without exfoliating the surrounding cells.

PIPAAm exhibits hydrophilic/hydrophobic alterations with external temperature changes, and cells on a PIPAAm surface can be collected by lowering the culture temperature from 37 to 20°C, while avoiding the use of digestive enzymes. Cells cultivated on this material flock together and form a monolayer sheet, thus enabling us to obtain a monolayer tissue that maintains intercellular adhesion (known as a cell sheet). In fact, cell sheets made from corneal epithelial cells are currently used for cornea regeneration [24], [25]. Therefore, this is also a nondestructive exfoliation method and is effective for collecting many cells. By contrast, our method is suitable for collecting single cells or small clusters of cells. For example, if there are several types of differentiated or undifferentiated cells derived from ES or iPS cells in a culture dish, our method enables the collection of only the targeted cells. Currently, ES and iPS cells are problematic in that they form tumors when transplanted into the living body [26], [27]. Our method ensures safe cell transplantation because of the ability to collect only differentiated cells. Furthermore, this method can be applied to the development of an imaging-based cell sorting system by recognizing cell shape, size, or cell cycle state, which is not possible with commercialized fluorescence-activated cell sorting (FACS) methods.

In conclusion, we have developed a new, non-destructive approach to cell preparation with phenotypic identification. The advantages of this method are that (1) cultured cell clusters or single cells can be collected non-destructively and (2) it is possible to control cell shapes in the preculturing stage and maintain those shapes during the reculturing process.
